# Pyoderma gangrenosum following anti-TNF therapy in chronic recurrent multifocal osteomyelitis: drug reaction or cutaneous manifestation of the disease? A critical review on the topic with an emblematic case report

**DOI:** 10.3389/fmed.2023.1197273

**Published:** 2023-05-31

**Authors:** Maurizio Romagnuolo, Chiara Moltrasio, Claudia Iannone, Maurizio Gattinara, Stefano Cambiaghi, Angelo Valerio Marzano

**Affiliations:** ^1^Dermatology Unit, Fondazione IRCCS Ca' Granda Ospedale Maggiore Policlinico, Milan, Italy; ^2^Department of Pathophysiology and Transplantation, Università degli Studi di Milano, Milan, Italy; ^3^Division of Clinical Rheumatology, ASST Gaetano Pini-CTO Institute, Milan, Italy; ^4^Department of Clinical Sciences and Community Health, Research Center for Adult and Pediatric Rheumatic Diseases, University of Milan, Milan, Italy; ^5^Pediatric Dermatology Unit, Department of Clinical Sciences and Community Health, Fondazione IRCCS Ca' Granda Ospedale Maggiore Policlinico, Milan, Italy

**Keywords:** pyoderma gangrenosum, chronic recurrent multifocal osteomyelitis, drug-induced PG, anti-TNF, adalimumab, adverse drug reaction, pustular lesion, pustular psoriasis

## Abstract

Chronic recurrent multifocal osteomyelitis (CRMO) is a rare autoinflammatory disease, clinically characterized by chronic and recurrent episodes of osteoarticular inflammation, that generally presents in children and adolescents. From a dermatological point-of-view, CMRO can be associated with skin rashes mainly including psoriasis, palmoplantar pustulosis and acne. Pyoderma gangrenosum (PG) is a rare immune-mediated inflammatory skin disease classified within the spectrum of neutrophilic dermatoses that, in some cases, has been reported as cutaneous manifestation in CMRO patients. This paper presents a 16-year female patient diagnosed with CMRO, who presented PG lesions located on the lower leg, that arose after the administration of the tumour necrosis factor (TNF)-α inhibitor adalimumab. Cases of PG have been reported in patients being treated with certain medications, including TNF-α antagonists, leading to classified them in a setting aptly termed “drug-induced PG.” In this paper, we discuss the co-occurrence of PG and CRMO, in the light of recent evidence on the pathogenesis of both diseases and giving ample space to a literature review on drug induced PG. In our case, it is plausible that PG could be considered a cutaneous manifestation of CRMO, although the mechanisms underlying this intriguingly relationship remain to be fully unraveled.

## 1. Introduction

Chronic recurrent multifocal osteomyelitis (CRMO) is a primary autoinflammatory bone disease that typically occurs in children and adolescents with a female to male ratio of 2:1, although the exact incidence and prevalence are not well-known (0.5–6/1,000,000 children) and are probably underestimated. It is clinically characterized by chronic and recurrent episodes of symptomatic, osteolytic/sclerotic bone lesions with a debilitating burden for skeletal and extra-skeletal system (1).

CRMO pathophysiology is multifactorial and includes a predisposing genetic background closely related to an unbalanced immune response as well as host-specific aspects and environmental influences (1).

CRMO can be associated, in addition to other conditions, with skin rashes mainly including psoriasis, palmoplantar pustulosis and acne (2).

Pyoderma gangrenosum (PG) is a rare, immune-mediated inflammatory skin disease belonging to the spectrum of neutrophilic dermatoses, clinically characterized by rapidly developing, painful skin ulcers hallmarked by undermined borders and peripheral erythema (3) that, in some cases, has been reported as cutaneous manifestation in CRMO patients (4). PG can either occur as an isolated dermatosis or in association with other immune-mediated diseases—most commonly inflammatory bowel diseases (IBD) and rheumatoid arthritis (RA)—, malignancies, or being a prominent clinical feature in the setting of monogenic autoinflammatory syndromes and related disorders (syndromic PG) (3, 5). The pathogenesis of PG is not fully understood, although a close interaction between genetic factors and immune dysregulation has been proposed (6).

In addition to systemic diseases, PG onset has been reported following the administration of different class of medications, including tumor necrosis factor (TNF)-α inhibitors, leading to a different class of PG termed “drug-induced PG,” itself an ideal model to better understand PG pathogenesis (7).

We describe a case of pyoderma gangrenosum in a 16-years-old girl that arose after the administration of the TNF-α inhibitor adalimumab for CRMO. Some of this case details have been previously reported by our rheumatology colleagues (8). Here, we illustrate the case from a dermatologic perspective, including clinical and histopathological description of PG lesions, and speculate on the possible nature of PG, whether consider it as a drug reaction or a cutaneous manifestation in the setting of CRMO, possibly exacerbated by the anti-TNF therapy.

The aim of the present article is to provide an up-to-date review on (i) the pathogenesis of the CRMO, (ii) the cutaneous manifestations associated to this rare disease and (iii) discuss the reported cases of drug-induced PG due to TNF-α inhibitors.

## 2. Case presentation

A 16-years-old girl affected by CRMO attended our dermatological department for the recent onset of gradually enlarging ulcerations on the lower legs. Previous treatment for CRMO included methotrexate 15 mg per week subcutaneously, nerindronate 2 mg/kg intravenously every 3 months and a 2-month cycle of naproxen 15 mg/kg per day. However, due to persistent skeletal involvement confirmed by magnetic resonance imaging (MRI), anti TNF-α agent adalimumab was introduced at the dosage of 40 mg every 2 weeks subcutaneously. Cutaneous lesions appeared after 2 months of the newly introduced adalimumab therapy and were reported at first as reddish papules, that suddenly enlarged and ulcerated (8).

**Figure 1:**
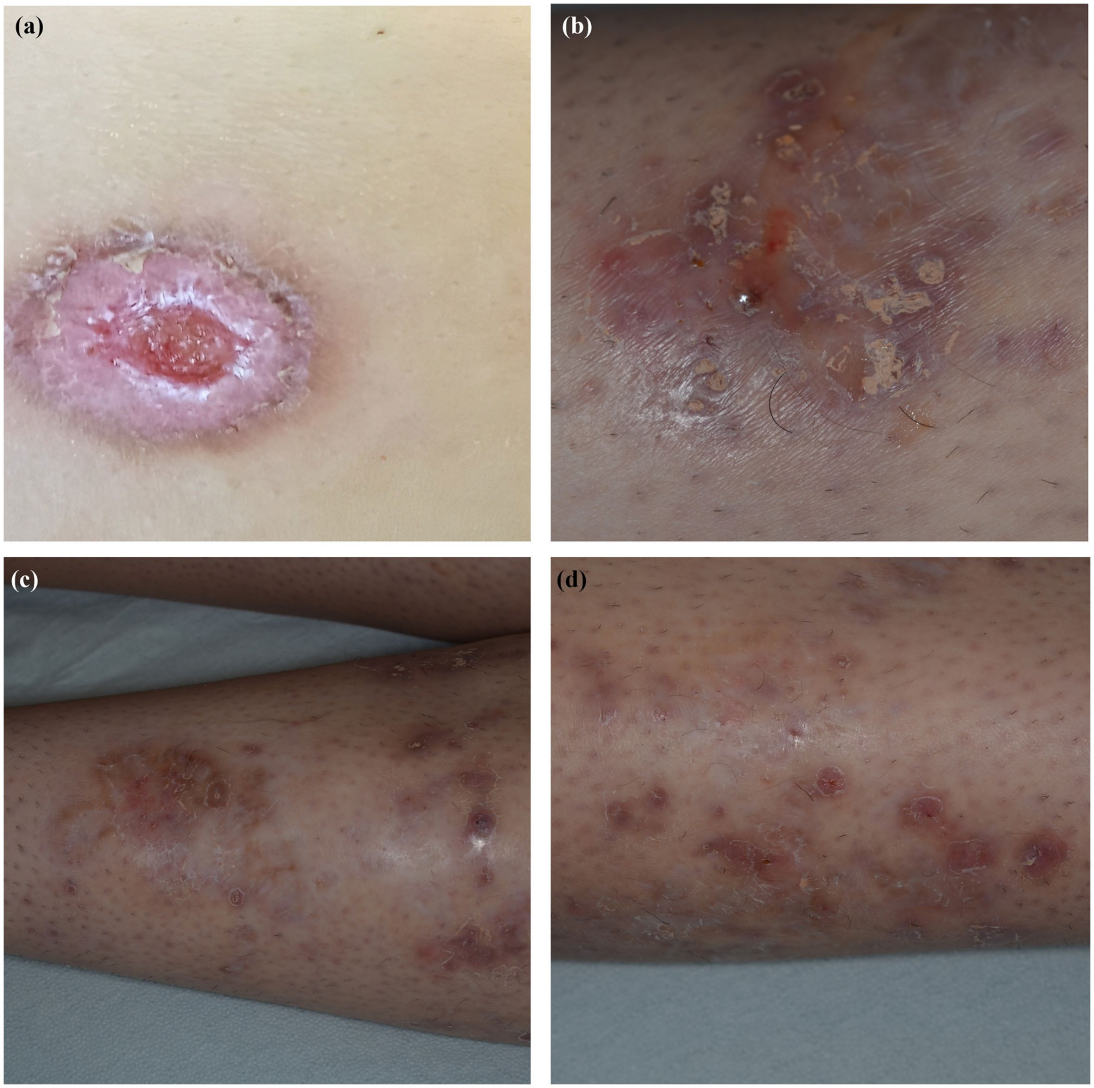
Clinical pictures showing **(A)** an elevated, violaceous plaque with an ulcerated center and crusted edges on the left leg of the patient; **(B)** after 3 months of therapy the lesions was substantially healed with a flat, atrophic surface and a wrinkled paper appearance; **(C,D)** a detail of the others atrophic and hyperpigmented sequelae resulting from previous erosive lesions on the left leg of the patient.

**Figure 2:**
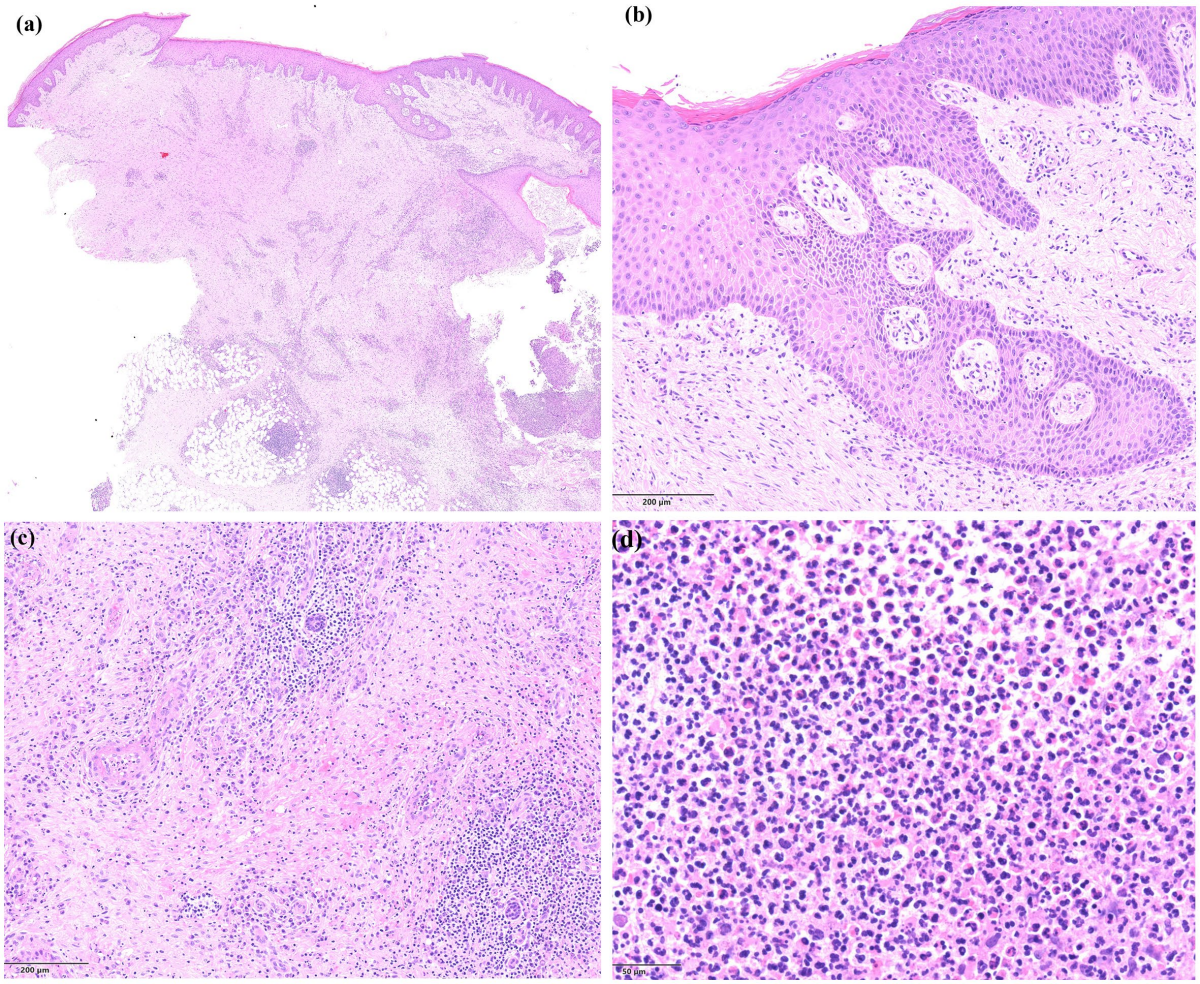
Histopathology of the lesions showing **(A)** an area of ulceration with cellular debris and neutrophils (on the right side), epidermal hyperplasia, and a dense inflammatory infiltrate with abundant neutrophils in the mid-to-deep dermis, together with extensive areas of necrosis and dilated blood vessels (haematoxylin and eosin staining, ×4); **(B)** a detail of the epidermal hyperplasia presenting with a pseudo-carcinomatous appearance (haematoxylin and eosin staining, ×10); **(C)** a detail of the dermal area of necrosis with dilated blood vessels and abundant inflammatory infiltrate (haematoxylin and eosin staining, ×10); **(D)** a close-up view of the dermal admixed inflammatory infiltrate, mainly composed of neutrophils (haematoxylin and eosin staining, ×20).

Dermatologic examination revealed a large, ulcerated lesion with elevated violaceous border and crusted edges on the left leg of the patient (Figure 1A). An incisional biopsy of the ulcerative lesion was performed for histopathological examination, that revealed a pseudo-carcinomatous epidermal hyperplasia, with an admixed inflammatory infiltrate mainly composed of neutrophils in the mid-to-deep dermis with areas of necrosis and reactive angiogenesis (Figure 2). The clinical and histopathological picture was consistent with a diagnosis of pyoderma gangrenosum.

PG onset was interpreted as a paradoxical reaction to adalimumab, which was withdrawn and switched with the anti-interleukin (IL)-1 agent canakinumab (4 mg/kg every 4 weeks subcutaneously), together with a 2-month cycle of systemic prednisone 15 mg per day and topical clobetasol twice a day. At the three months dermatological follow-up the lesions were partially healed and appeared as multiple irregular erosions with a crusted and scaly surface located on the left leg of the young patient; the bigger lesions healed with a sclerotic and atrophic retracting scar tissue with a “wrinkled paper” appearance (Figures 1B–D).

Canakinumab schedule was reduced to 4 mg/kg every 8 weeks but 1 year after complete clinical remission CRMO relapsed with bone pain, and new pustular and ulcerative lesions on the lower legs appeared, readily treated with systemic corticosteroids and re-scheduling canakinumab every 4 weeks (4). During the last follow-up, which took place 8 months after the last therapeutic changes, and approximately 3 years and 8 months after the onset of CRMO, the patient was no longer taking corticosteroids and was only receiving canakinumab every 4 weeks. She was in good general condition, and her skin lesions had completely healed with cribriform scarring.

From a dermatologic perspective the onset of a PG or PG-like lesions during anti-TNF therapy often pose a diagnostic and therapeutic challenge, as it is hard to discriminate between a natural history of the disease (in most cases patients use the anti-TNF agent for diseases classically associated to PG) or an adverse “paradoxical” reaction to this class of drug, as TNF inhibitors represent a therapeutic option for PG itself (9).

In our case a Naranjo score of 3 was calculated, indicating a possible adverse drug reaction to adalimumab. However, a flare up of pustular and erosive lesions was also reported during canakinumab treatment and is possible, considering the autoinflammatory nature of both conditions, that PG lesions could belong to the spectrum of neutrophilic dermatoses associated to CRMO. In the next sections, we provide a focused review on the topic based on the published literature.

## 3. CRMO etiopathogenesis

Despite recent scientific achievements, the exact molecular pathophysiology of CMRO remains unclear. However, it is believed to be related to a close interaction between genetic, host-specific aspects and environmental influences that contribute to the susceptibility, onset, severity, and clinical progression of this disease. Furthermore, an altered immune system response leading to chronic inflammation has been reported to play a pivotal role with upregulation of diverse proinflammatory cytokines and other effectors molecules.

### 3.1. Immune network

The pathomechanisms that orchestrate the cell type- and receptor-specific induction of cytokines and chemokines, in most cases, have yet to be elucidated. CRMO in humans can be modelled using mouse models carrying a missense mutation (Leu98Pro) in the *PSTPIP2* (Proline-Serine–Threonine Phosphatase Interacting Protein 2) gene (10), a member of the Pombe Cdc15 homology (PCH) family of proteins selectively expressed in macrophages and macrophage precursors, that has been shown to coordinate membrane and cytoskeletal dynamics. This led to the identification of Interleukin (IL)-1β and its receptor as key drivers of bone lesions in CRMO, highlighting the “redundant” role for NLRP3 (NLR family pyrin domain containing 3)/caspase-1 and caspase-8. Although this latter is mostly known for play a central role in the execution-phase of cell apoptosis, its activation also promotes the cleavage of IL-1β in CRMO pathogenic context (11). To investigate the factors upstream of caspase-8 activation, Dasari et al. (12), using several mutant mouse strains, immunoblotting, and microcomputed tomography, first discovered a key role of the nonreceptor tyrosine kinase SYK (spleen tyrosine kinase) in mediating CRMO. Indeed, in *Pstpip2^cmo^* mice carrying a genetic deletion of *Syk*, the disease phenotype was completely abrogated, revealing that SYK mediates signaling pathways upstream of caspase-1 and caspase-8 activation and up-regulates NF-κB (nuclear factor kappa-light-chain-enhancer of activated B cells) and IL-1β signaling, thus inducing CRMO phenotype.

Despite the well-recognized role of IL-1β, CRMO also results from an imbalance between several other pro- and anti-inflammatory cytokines (13). It has been underlined that CRMO patients show a decreased production of anti-inflammatory cytokines, such as, IL-10, IL-18 and IL-19, and an increased production of proinflammatory mediators including, among others, the aforementioned IL-1β, IL-6, IL-20 and TNF-α (13–17). Specifically, it has been demonstrated that monocytes from patients with CRMO do not express IL-10 in response to stimulation of the TLR (Toll-like receptor) 4 by LPS (lipopolysaccharides) (15) due to a defect in activation of ERK1 and ERK2 Map Kinases (18). In contrast, the proinflammatory pathways, mediated by Jun kinase (JNK) and p38MAPK, function normally.

As mentioned above, the pro-inflammatory cytokines, TNFα, IL-1β, IL-6, and IL-20 are overexpressed and not balanced by anti-inflammatory mediators, thus promoting an enhanced proinflammatory state. It has been assumed that this pathogenic scenario may result in an increased interaction between RANK (receptor activator of nuclear factor-κB) and its ligand RANKL on osteoclast precursor cells, thereby inducing osteoclast differentiation and activation (13, 19, 20).

A recent work conducted by Srdjan Grusanovic et al. (21), indicated that the CRMO phenotype can be summarized in two distinct mechanisms: (i) the IL-1β/MyD88 signaling pathway and (ii) the JAK/STAT3 (Janus kinase/signal transducer and activator of transcription 3) pathway, in part activated by IL-6, and responsible for HSC (hematopoietic stem cells) alterations. Indeed, these authors demonstrated that the CMRO environment possesses a myeloid gene signature that establishes a pro-inflammatory profile on HSCs, mainly driven by IL-6 and Jak/Stat3 signaling pathway; however, while IL-6 and Stat3 blockage reduces HSC numbers in CMRO mouse models, only inhibition of STAT3 activity significantly rescues their fitness, paving the way for novel therapeutic targets (STAT3 inhibitors) able to preserve HSCs activities.

### 3.2. Genetic factors

In addition to the molecular pathomechanisms mentioned above, gene defects have also been identified in the pathogenesis of CRMO: IL-10 expression is influenced by genetic variants within the IL-10 proximal promoter region; in particular, three promoter haplotypes (rs1800896, rs1800871, and rs1800872) affect the capacity of the IL-10 promoter to recruit the zinc finger transcription factor Sp-1 (22). Moreover, since IL-10 induces activation of STAT3, it has been demonstrated that individuals carrying IL-10 promoter haplotypes coding for “low” IL-10 expression, may in turn contribute to reduce STAT3 activation with consequent effects on FBLIM1 (Filamin Binding LIM Protein 1) expression (23). This latter is involved in the assembly and stabilization of actin-filaments and likely plays a role in modulating cell adhesion, cell morphology and cell motility, acting as an anti-inflammatory mediator that controls bone remodelling through the regulation of RANKL activation via ERK1/2 phosphorylation (24). Homozygous and compound heterozygous pathogenic variants in the *FBLIM1* gene were detected in unrelated CRMO patients (24, 25), confirming a potential causative role of this gene. However, its effective role in CRMO pathogenesis is still debated (26).

Yahara et al. indicated that the interaction between some haplotypes of killer cell immunoglobulin-like receptors (KIR), key regulators of human natural killer cell function, and the human leukocyte antigen (HLA) may be critical in the CRMO onset, leading to HLA instability that in turn leads to autoimmunity in this disease (27). The role of HLA was also confirmed by a genomic expression profiling and bioinformatic analysis in a CRMO cohort (28), together with other immune-related genes including, among other, *IRF5* (interferon regulatory factor 5) and *OAS3* (2′-5′-Oligoadenylate Synthetase 3) as well as several ribosomal-related genes. In addition, the same authors showed the impairment of oxidative phosphorylation, ribosome, and parkinson disease signaling pathways in CRMO patients (28).

Whole exome sequencing study conducted on 99 CRMO patients including 88 trios, identified 13 patients with heterozygous sequence variants in *FGR* gene—a member of Src family tyrosine kinases (SFKs)—, two of which with missense mutations in the C-terminal region of this gene. Functional studies demonstrated that missense pathogenic variants in this region amplified the inflammatory phenotype, indicating that gain-of function mutations in *FGR* may be involved in the CRMO pathogenesis (29), supporting a pronounced and unbalanced inflammatory network.

Finally, a recent blood transcriptomics study conducted by Ha et al. (30), revealed that innate immune responses including myeloid leukocyte and granulocyte activation, neutrophil activation and degranulation play a pivotal role in the pathophysiology of CRMO, together with a strong cellular response to cytokines.

It’s fair to mention two other genes most involved in familial/monogenic forms of CRMO: *LPIN2* and *IL1R.*

Some CRMO patients have been shown to have pathogenic variants in the *LPIN2* (Lipin 2), a protein coding gene that acts during normal adipose tissue development, playing a role in human triglyceride metabolism as a phosphatidate phosphatase enzyme, magnesium dependent. Loss-of function mutations in this gene are related to Majeed syndrome, clinically characterized by early-onset multifocal osteomyelitis, congenital dyserythropoietic anemia, and joint contractures. The disruption of the phosphatidic acid phosphatase activity in *LPIN2* results in immune dysregulation with subsequent overproduction of proinflammatory cytokines including IL-1β (31).

There is also DIRA (Deficiency of IL-1 Receptor Antagonist), a rare life-threatening autoinflammatory disease caused by autosomal recessive pathogenic variants in *IL1RN* gene (32); is clinically characterized by early onset generalized pustulosis, multifocal osteomyelitis within the first few postnatal weeks, and elevation of acute phase reactants. It has been demonstrated that the *IL1RN* gene modulates a variety of IL-1 related immune and inflammatory responses and, when dysregulated, drives an unchecked inflammation.

### 3.3. Microbiota: the “rising star”

The growing interest in the involvement of host-associated microbiota in various diseases has brought attention to this topic in CRMO as well. Rausch et al. (33) showed, among other findings, an interesting association involving the HACEK group (*Haemophilus parainfluenzae, Aggregatibacter actinomycetemcomitans, A. aphrophilus, A. paraphrophilus, Cardiobacterium* spp.*, Eikenella corrodens, Kingella spp*.) and disease activity.

Members of the HACEK group are normal indigenous flora of the oral cavity, and they are involved in unusual infections with cardiac and skeletal sequelae. *Eikenella* is well known to cause osteomyelitis and endocarditis (34), while *Kingella* has been recently recognized as a widely distributed pathogen for children, with disease manifestations in joints and bones (35).

*Aggregatibacter* can disrupt the membrane integrity of neutrophils *via* leukotoxin A (LtxA), thereby affecting their proper functions (36), as well as to induce prostaglandin, TNFα, and IL-6 expression (37), central drivers of CRMO pathogenesis.

*Cardiobacterium* is also thought to increase IL-1β levels (38) as well as *Actinomyces* (39) and *Cutibacterium acnes*. This latter was isolated from biopsies of CMRO and SAPHO patients (with simultaneous skin manifestations), suggesting its potential pathogenic role in these conditions, mainly due to a genetic predisposition for its altered clearance (40). However, its role is still controversial.

In conclusion, HACEK bacteria together with *Corynebacterium, Actinomyces, Propionibacteria, and Leptotrichia*, generally associated to infections that can develop into local and systemic inflammation with bone involvement, may promote a disease-modulating community that lead to CRMO phenotype (33).

Despite the reported evidence on CRMO pathogenesis, further studies are necessary to unravel the exact mechanisms underlying this condition.

## 4. CRMO: clinical manifestation and diagnosis

CRMO is a chronic autoinflammatory disease characterized by osteolytic, hyperostotic, and osteosclerotic lesions mainly affecting the metaphysis of long bones.

Overall, girls and young women appear to be more frequently affected (approximately 2:1), and most patients develop symptoms between 7 and 9 years-of-age. Inflammatory bone lesions may be unifocal or multifocal. Except for the neurocranium, which is almost never affected, inflammation can affect all sites of the skeleton (41). Typical sites of inflammation include the clavicle and the sternum, and rarely the mandible. Clinically silent bone lesions can occur and may affect vertebral bodies, arthritis may be present near the affected bones, as well as in other sites. In a few cohorts, a strong clinical link towards spondylarthritis (SPA) has been noted (42). CRMO onset is usually insidious, and inflammatory bone pain shows a relapsing–remitting course. Systemic features, such as fever, night sweats and/or weight loss may occur in up to 17% of children and may raise concerns about alternative diagnoses (43). Other organ systems may be involved as well. Up to 20% of the patients develop skin manifestations, including palmoplantar pustulosis, acne and psoriasis (44). Chronic inflammatory bowel disease may be present in up to 10% of patients (45, 46). Occasionally, hepatosplenomegaly and lymph node enlargement are noted (up to 3% of patients). Ocular or cardiac manifestations are generally rare.

As no specific biomarkers are currently available, CRMO remains a diagnosis of exclusion as widely accepted and prospectively tested diagnostic criteria have not yet been established. Indeed, malignancies including osteosarcoma, Ewing sarcoma, leukemia, lymphoma, skeletal metastases, or systemic diseases such as Langerhans cell histiocytosis are required to be ruled out before suspecting CRMO. Additionally, infections, trauma, and other autoimmune or inflammatory bone diseases must also be considered when making a differential diagnosis of CRMO. It is recommended to test for infectious agents the fresh bone tissue, including the search for mycobacteria. Laboratory test classically show a mild to moderate increase of C-reactive protein (CRP) and erythrocyte sedimentation rate (ESR).

Imaging plays a pivotal role in the diagnosis of CRMO. Bone lesions appear as radiolucent, osteolytic, or sclerotic areas on plain radiographs; however, during the early stages, they may be undetectable. Thus, conventional radiology is almost always followed by whole body imaging, which is a pivotal tool for the diagnosis of CRMO and is performed to monitor for additional sites of bone inflammation, especially in the vertebral column. Additionally, whole body-magnetic resonance imaging (WB-MRI) represents the gold standard to screen for potentially clinically silent lesions and mark their evolution.

Bone biopsy is still considered the gold standard for diagnosis, despite being WB-MRI most used in clinical practice. Findings in bone biopsies from CRMO patients include dense infiltrates of immune cells, lysis of bone, fibrosis and/or normal bone. Indeed, cellular infiltrates can change over time with mainly innate immune cells in early disease stage. Macrophages are to the most stable cellular component during the different disease phases. However, innate and adaptive cellular infiltrates can frequently coexist (47, 48).

## 5. Cutaneous manifestations associated to CRMO

Cutaneous involvement in CRMO patients has been invariably reported since the first case descriptions (49–51); in particular, the strong association with palmoplantar pustulosis (PPP) led to consider PPP and/or psoriasis as one of the major diagnostic criteria for the CRMO basing on the 2007 Jansson classification (52).

More recently, an epidemiological analysis from the Eurofever registry on 486 CRMO cases revealed that 19% of patients reported mucocutaneous manifestation of which: 5% presented acne, 5% PPP, 4% psoriasis, 3% papulopustular lesions, 2% urticaria and 1% aphthous stomatitis (44). Even though the severity of the skin condition was not reported in the former analysis it is striking—apart from acne which is commonly more frequent in the youth population—the predominance of PPP, psoriasis, and papulo-pustular manifestations in the studied population. At the same time, considering the intrinsic neutrophils-driven autoinflammatory nature of CRMO, it is not surprising that cutaneous manifestation belonging to the spectrum of neutrophilic dermatoses could be associated to this rare disease. To further support this hypothesis, in addition to acne and psoriasis, cases of Sweet syndrome, PG, acne fulminans and pathergy phenomena were also reported as dermatological manifestations in CRMO patients (4). Focusing on PG or PG-like lesions, up to date 8 cases were reported in the literature in association with CRMO (Table 1) (53–60). Consistently with the epidemiological data of the CRMO most of the patients were female (6/8) and children/adolescents (median age 13 years, range 1–26 years). In 6/8 cases PG onset followed the diagnosis of CRMO, while in two cases cutaneous and bony lesions appeared almost synchronously (59, 60); lower legs, including the dorsum of the foot was documented in 6/8 cases PG cases, while in the two remaining cases PG lesions were located on the arms; no “atypical” locations such as face, genitalia, or trunk involvement was reported (Table 1). Classical ulcerative PG of the anterior aspect of the lower leg was reported in two cases (54, 56), while in other cases different PG forms, including vegetative and superficial variants were described (53, 55, 57). In the case presented by Wurm et al. multiple superficial pustular lesions on the lower legs were reported (58). Interestingly, in half of the reported cases (4/8) PG ulceration was located in the same anatomical location of the bone inflammation: in three cases PG outbreak on the skin overlying bone biopsy site suggesting a pathergy phenomenon (54, 55) while in the case reported by Katsuo et al., PG was contiguous with the site of bone inflammation on the foot of a 2-years-old boy (60). Regarding the former case, the patient was under therapy with the TNF inhibitor etanercept, which was not discontinued due to the good clinical course of CRMO, and the cutaneous and contiguous bony lesions improved with a cycle of steroids; it was the only case in the series in which anti TNF agents were employed for the CRMO (Table 1). In all the reported cases PG lesions resolved with conventional immunosuppressive and/or anti-inflammatory therapy.

**Table 1:** Clinical overview of the reported PG cases occurring in CRMO patients.

Author, year/reference	Sex, age	PG location	PG characteristics	PG treatment	PG outcome	Comorbidities
Sundaram M. et al., 1996/(53)	F, 10	Right leg, in the previous bone biopsy site	ND	Oral CCS	CR	–
Omidi C.J. et al., 1998/(54)	F, 12	Right tibia (bone biopsy site)	Classical ulcerative	Intravenous CCS	CR	UC
Nurre L.D. et al., 1999/(55)	F, 3	Left arm, bone biopsy site	Bullous, superficial	Prednisone 2 mg/kg/die	CR	–
Williamson D. et al., 2002/(56)	F, 26	Right leg	Classical ulcerative	- Topical wound therapy - Intralesional CCS - Sulfasalazine	CR	Plantar pustular psoriasis
Koturoğlu G. et al., 2006/(57)	M, 6 months	Right arm, legs	Bullous, superficial	Prednisone 2 mg/kg/die	CR	–
Wurm M.C. et al., 2016/(58)	F, 14	Lower legs	Multiple superficial ulcerations	- Ibuprofen - Antibiotics	CR	–
Dagan O. et al., 2017/(59)	F, 10	Left foot	Vegetative	- Prednisolone 1 mg/kg/die - Mycophenolate mofetil	CR	Takayasu arteritis
Katsuo K. et al., 2020/(60)	M, 2	Left foot, underlying bone lesion	Subcutaneous nodule with ulceration	Etanercept (5 mg/kg twice weekly)	CR	–

PG, pyoderma gangrenosum; CRMO, chronic recurrent multifocal osteomyelitis; F, female; ND, no data; CCS, corticosteroids; CR, complete remission; UC, ulcerative colitis; M, male.

## 6. Anti TNF drug-induced PG

Certain drugs and class of medications have been reported to trigger and/or exacerbate pyoderma gangrenosum, taking into consideration the hypothesis that some cases of PG could be categorized as drug induced. Even though a direct physio-pathological link is difficult to establish in such cases, cytokine imbalance, dysregulated neutrophil migration and function, keratinocyte apoptosis and epigenetic factors, all of which could be modified by the drugs effect, could play a role in the pathophysiological scenario of drug-induced PG, as elegantly reviewed by Wu et al. (7). Regarding TNF-α inhibitors, the unbalanced hyperproduction of interferon gamma by plasmacytoid dendritic cells and CD4+ lymphocytosis derived from TNF-dependent decreased apoptosis have been suggested as key drivers of PG onset in predisposed individuals (3). These reactions are defined paradoxical as TNF-α inhibitors are currently used as second-line treatment in PG refractory to conventional immunosuppressive therapy (61, 62).

To the best of our knowledge 13 additional cases of PG during anti TNF-α therapy have been reported in the literature (Table 2) (8, 63–75). All the licensed anti TNF-α agents (etanercept, adalimumab, infliximab, golimumab and certolizumab) have been reported in association to drug-induced PG; nearly half of the cases (7/13) were due to adalimumab (monoclonal antibody binding soluble and membrane bound TNF-α). Most of the reported reactions occurred in middle-aged individuals (median age 56, range 34–82 years) and within 6 months of the anti TNF-α agent introduction (Table 2). In all the cases, TNF-α inhibitors were prescribed for diseases which takes part in the comorbidity spectrum of classical and/or syndromic PG, such as rheumatoid arthritis (6/13), ulcerative colitis (2/13), psoriatic arthritis (3/13), ankylosing spondylitis (1/13) and hidradenitis suppurativa (1/13). The clinical presentation of drug-induced PG in the reported cases was variable, although in nearly half of the cases (6/13) ulcerations were present in different body areas (multifocal PG), with the lower portion of the legs as a commonly involved site (8/13). Interestingly, in some cases PG onset was accompanied by extracutaneous symptoms such as fever and anterior uveitis (72), pyogenic arthritis of the left knee (71), and bilateral sacroiliitis along with psoriasiform palmar eruption (69). A paradoxical skin reaction characterized by an overlap between pustular psoriasis and PG lesions was also reported (74).

**Table 2:** Overview of the drug-induced PG cases reported in the literature.

Author, year/reference number	Anti-TNF α agent	Sex, age	Primary condition	PG onset (months after treatment initiation)	PG location(s)	Discontinuation of the TNF-inhibitor	Therapeutic intervention	Outcome
Vandevyvere K. et al., 2007/(63)	Infilximab	F, 53	RA	ND	Left foot	Yes	- Minocycline 100 mg/die - Methylprednisolone 4 mg/die - Cyclosporine 3 mg/kg/die - Etanercept	CR
Stichenwirth M. et al., 2008/(64)	Adalimumab	M, 42	RA	18	Right cheek; left arm; right shoulder	Yes	- Methylprednisolone 1 mg/kg/die - Ciclosporine 5 mg/kg/die	CR
Jaimes-Lòpez N. et al., 2009/(65)	Infliximab	M, 47	UC	1	Multiple: trunk; abdomen; legs; peristomal area; face; genitalia	Yes	- Prednisone 1 mg/kg/die - Topical tacrolimus	PR, expired after 7 months due to sepsis
Brunasso A.M. et al., 2010/(66)	Infliximab	F, 40	UC	<1 (2 weeks)	Pretibial area, bilaterally	Yes	Cyclosporine 5 mg/kg/die	CR
Kikuchi N. et al., 2012/(67)	Adalimumab	F, 50	RA	3	Legs, bilateral	Yes	Prednisone 40 mg/die	CR
Kowalzick L. et al., 2013/(68)	Etanercept	F, 58	PsA	1	Left temple; right groin; pubic area	Yes	Prednisone 1 mg/kg/die	CR
Mir-Bonafè J.F. et al., 2017/(69)	Adalimumab	M,82	RA	3	Breast, bilateral	Yes	Ciclosporine 200 mg/die	CR
Benzaquen M. et al., 2017/(70)	Adalimumab	F, 56	PsA	6	Right leg	Yes	Secukinumab	CR
Skalkou A. et al., 2017/(71)	Golimumab	F, 55	RA	12	Left leg and foot, multiple lesions	Yes	- Prednisone 1 mg/kg/die - Cyclosporine - Surgical debridement	CR
Fujimoto N. et al., 2018/(72)	Infliximab	F, 48	PsA	ND	Right leg	Yes	Adalimumab	CR
Company-Quiroga J. et al., 2019/(73)	Adalimumab	M. 50	HS	1	Left leg (multiple lesions)	Yes	Methylprednisolone 1 mg/kg/die	CR
Gawdzik A. et al., 2020/(74)	Certolizumab	F, 34	AS	6	Right breast; pubic region; left foot; right tight;	Yes	-Secukinumab - Methotrexate 20 mg/week	CR
Tan Y. et al., 2021/(75)	Adalimumab (biosimilar)	M, 48	RA	<1 (2 weeks)	Dorsal hand, bilateral	Yes	Etanercept (biosimilar)	CR
Acierno S. et al., 2022/(8) (our case)	Adalimumab	F, 16	CRMO	2	Legs, bilateral	Yes	- Prednisone 20 mg/die - Clobetasol ointment twice a day - Switch to canakinumab	CR; recurrence after 1 year

TNF α, tumor necrosis factor-α; PG, pyoderma gangrenosum; F, female; RA, rheumatoid arthritis; ND, no data; CR, complete remission; M, male; UC, ulcerative colitis; PR, partial remission; PsA, psoriatic arthritis; HS, hidradenitis suppurativa; AS, ankylosing spondylitis; CRMO, chronic recurrent multifocal osteomyelitis.

In all the reported cases the TNF-α agent was withdrawn and a systemic immunosuppressive therapy was initiated with complete remission of the skin lesions, except for a patient who expired after 7 months for a sepsis (65). Of note, in 3 cases the culprit drug was switched with another anti-TNF agent, with no evidence of PG relapses (63, 72, 75); however long-term follow-up data were not available for most of the reports.

Anti TNF-α cutaneous paradoxical reaction are rare events, mainly characterized by psoriasiform eruption (especially palmo-plantar and pustular lesions) in up to 75% of the cases, and to a lesser extent by sarcoidosis and HS (9, 76). A recent review on drug-induced PG, basing on the Naranjo algorithm, critically analysed the role of different class of drugs in the dermatosis onset, concluding that anti-TNF agents were weakly associated to drug-induced PG (mean Naranjo score between 1 and 4, indicating only a possible adverse reaction) (77). Our case presentation is in line with the former data as the calculated Naranjo score was 3; moreover, the re-appereance of the pustular lesions even during anti TNF-α discontinuation, indicates that PG onset was probably linked to CRMO, while adalimumab represented an additional risk factor. In addition, the role of canakinumab in controlling the skin disease remains controversial due to the concomitant administration of systemic corticosteroids during the PG flare-up.

## 7. Conclusion

The relationship between pyoderma gangrenosum and CRMO remains controversial, although considering the recent advances in understanding the pathogenetic context of both diseases, hallmarked by a neutrophil-mediated inflammation, and the reported association between CRMO and neutrophilic reactive dermatoses, it is plausible that PG could be considered a cutaneous manifestation of CRMO.

On the other hand, it is known that anti-TNF therapies could induce and/or aggravate neutrophilic diseases, including PG, acting as additive risk factors for the onset of such manifestations in predisposed individuals.

Basing on the current data, it seems reasonable in CRMO setting (i) to withdraw the anti-TNF agent if PG or PG-like pustular eruptions appear following the newly introduced therapy (especially within the first 6 months of treatment), (ii) administer a cycle of conventional immunosuppressants (corticosteroids and/or cyclosporine) and (iii) consider to switch the TNF inhibitor with another effective biologic agent.

Children and young adults presenting with ulcerative and pustular lesions (including pustular psoriasis and its variants) who complain of chronic and recurrent bone pain should promptly undergo an accurate clinical and, if advised, radiological examination to exclude CRMO. In this context dermatologist could play a crucial role in an early diagnosis with a better clinical management of this rare and challenging disease.

## Data availability statement

The original contributions presented in the study are included in the article/supplementary material, further inquiries can be directed to the corresponding author.

## Author contributions

MR and CM contributed to conception and design of the study. MR, CM, and CI wrote the first draft of the manuscript and performed the literature search. MG, SC, and AM supervised the work. All authors contributed to the article and approved the submitted version.

## Funding

This research was partially supported by the Italian Ministry of Health (Ricerca Corrente 2023), Fondazione IRCCS Ca’ Granda Ospedale Maggiore Policlinico, Milan (Italy).

## Conflict of interest

The authors declare that the research was conducted in the absence of any commercial or financial relationships that could be construed as a potential conflict of interest.

## Publisher’s note

All claims expressed in this article are solely those of the authors and do not necessarily represent those of their affiliated organizations, or those of the publisher, the editors and the reviewers. Any product that may be evaluated in this article, or claim that may be made by its manufacturer, is not guaranteed or endorsed by the publisher.
